# A fuzzy method for RNA-Seq differential expression analysis in presence of multireads

**DOI:** 10.1186/s12859-016-1195-2

**Published:** 2016-11-08

**Authors:** Arianna Consiglio, Corrado Mencar, Giorgio Grillo, Flaviana Marzano, Mariano Francesco Caratozzolo, Sabino Liuni

**Affiliations:** 1 Institute for Biomedical Technologies of Bari - ITB, National Research Council, Bari, 70126 Italy; 2 Department of Informatics, University of Bari Aldo Moro, Bari, 70121 Italy

**Keywords:** RNA-Seq, Differential expression, Multireads, Fuzzy sets, Possibilistic modeling

## Abstract

**Background:**

When the reads obtained from high-throughput RNA sequencing are mapped against a reference database, a significant proportion of them - known as multireads - can map to more than one reference sequence. These multireads originate from gene duplications, repetitive regions or overlapping genes. Removing the multireads from the mapping results, in RNA-Seq analyses, causes an underestimation of the read counts, while estimating the real read count can lead to false positives during the detection of differentially expressed sequences.

**Results:**

We present an innovative approach to deal with multireads and evaluate differential expression events, entirely based on fuzzy set theory. Since multireads cause uncertainty in the estimation of read counts during gene expression computation, they can also influence the reliability of differential expression analysis results, by producing false positives. Our method manages the uncertainty in gene expression estimation by defining the fuzzy read counts and evaluates the possibility of a gene to be differentially expressed with three fuzzy concepts: over-expression, same-expression and under-expression. The output of the method is a list of differentially expressed genes enriched with information about the uncertainty of the results due to the multiread presence.

We have tested the method on RNA-Seq data designed for case-control studies and we have compared the obtained results with other existing tools for read count estimation and differential expression analysis.

**Conclusions:**

The management of multireads with the use of fuzzy sets allows to obtain a list of differential expression events which takes in account the uncertainty in the results caused by the presence of multireads. Such additional information can be used by the biologists when they have to select the most relevant differential expression events to validate with laboratory assays. Our method can be used to compute reliable differential expression events and to highlight possible false positives in the lists of differentially expressed genes computed with other tools.

**Electronic supplementary material:**

The online version of this article (doi:10.1186/s12859-016-1195-2) contains supplementary material, which is available to authorized users.

## Background

The advent of High-performance Next-Generation Sequencing (NGS) technologies has improved the analysis of differential expression of genes, by investigating both the nature and the quantity of expressed mRNAs and increasing the spectrum of applications of sequencing.

Increasingly faster mapping algorithms and more complex statistical analyses have been developed to address all the issues introduced by RNA-Seq data, but the discussion about the best workflows is still open.

A typical differential expression (DE) analysis workflow is composed by three main steps: (1) read mapping, (2) gene expression computation and (3) identification of noticeable differences between the samples. The results are generally output as a list of genes showing a statistically significant variation in expression between different experimental conditions. The confidence of the result is usually defined through fold change values and *p*-values obtained with statistical hypothesis testing.

Despite the continuous improvement of the sequencing techniques, the results of DE analyses are not yet fully reliable. The variations in gene expression discovered from RNA-Seq data must be confirmed with laboratory assays, such as qPCR and these validations often reveal the presence of false positives in the bioinformatics analysis results [1]. In order to obtain more accurate results, the second and third steps of DE analysis workflow have been deeply examined in the last years, also taking advantages from the experience gained with the use of microarrays. Several normalization techniques [2–4] and DE analysis models [5–7] have been designed for sequence count data.

More recently some attention has been focused on an issue that arises in the first step of DE analysis workflow, i.e. the problem of multireads, which are those reads that map to more than one transcript/genomic location in the reference sequences and cause uncertainty in gene counts.

The main source of mapping ambiguity is the presence of genes with similar sequences (i.e., paralogous gene families) and, since the sequenced reads do not span entire transcripts, alignment algorithms are sometimes unable to uniquely determine the gene from which they are derived. In addition, polymorphisms, inaccurate reference sequences and sequencing errors require mismatches and indels to be allowed in read alignments, lowering out the quality of the mapping process.

When the number of multireads is small, many researchers simply choose to exclude such reads from the analysis, counting only uniquely mapping reads [8], but this option always leads to an underestimation of the read counts and it is not possible to a priori know if the removed reads were relevant for the DE results.

Another approach is to estimate the real number of read counts: the simplest way is to assign multireads to genes proportionally to the expression of uniquely mapping reads (named Rescue Method) [9]; some more complex techniques compute an estimation of the read counts using probabilistic models, such as IsoEM [10], RSEM [11], Rcount [12], TEtranscript [13], MMR [14]. These methods, starting from some assumptions on the distribution of the data, model the generation of multireads and estimate the final read counts. From each method we obtain different estimated counts, and their confidence value, when provided, is not considered by the actual DE analysis tools and it cannot be used to evaluate the results. In a recent work, Robert and Watson [15] propose a two stage analysis: in stage 1, reads are assigned uniquely to genes; in stage 2, reads that map to multiple genes are assigned uniquely to multi-map groups, but 30 % of such groups contains two or more genes. The expression variation of a single gene could either be hidden in the group, or it could require a laboratory validation of all the genes in the group.

In this paper, starting from an approach for dealing with multireads that preserves uncertainty information, we present a novel DE analysis workflow. The main aim of our work is to extract the results with high possibility of being true positives, and hence to highlight those results that risk to be false positives, since their count values are influenced by the presence of multireads. The whole workflow we describe is based on fuzzy set theory.

Fuzzy sets were introduced by Zadeh as a mean to model imprecisely defined concepts in 1965 [16], by handling partial truth with the concept of gradual membership to a set. Fuzzy logic is a generalization of classical logic based on fuzzy set theory. A fuzzy set could be used to restrict the possible values a variable can assume, thus providing a possibility distribution for the variable [17].

Possibility theory is the theory of handling possibility distributions; it was introduced by Zadeh and further developed by Dubois and Prade [18]. Possibility theory and probability theory are only loosely related, and the former seems more suitable to model uncertainty deriving from imprecision or lack of information.

The approach used in this paper is compliant with the work of Zadeh [17], who proposed possibility distributions as suitable interpretations of fuzzy sets. The possibility measure used in this paper follows the notation introduced by Pedrycz [19].

The method has been tested on public RNA-Seq data and discussed by comparing the obtained results with other bioinformatics tools. The read count estimations have been computed with TopHat and Bowtie mappings and with RSEM. The DE analyses were performed on the counts with cuffdiff, DESeq2, edgeR and with Fisher’s Exact test adjusted by false discovery rate (FDR).

## Methods

Our workflow starts with the quantification of gene expressions in presence of multireads through the definition of fuzzy read count. It applies fuzzy sets to build a granular representation of read counts for each gene. A whole new workflow is then introduced to extend DE analysis to this expression measure.

For this purpose, we define the fuzzy fold change, the possibility of over or under-expression and the possibility of same-expression, that is useful to evaluate the presence of false positives in the DE events. The output of the workflow is a list of DE events with the additional information about uncertainty correlated to multiread presence.

### Fuzzy description of count data

When the NGS reads are compared to a database of reference sequences such as genes or transcripts, we obtain a list of results as summarized by Table 1. The mappings among reads and genes are provided one by row and the multiread events are represented by multiple rows identified by the same read. If mismatches and indels are allowed in the mapping results we can obtain, for the same read, matches that differ in mapping accuracy.

**Table 1:** Example of mapping results with multireads

Read ID	Reference ID	Identity	Coverage
read-1	gene-1	100	100
read-2	gene-1	100	100
read-2	gene-2	99	80
read-2	gene-3	95	80
read-3	gene-1	100	100
read-3	gene-2	100	100
read-4	gene-1	96	90
read-4	gene-2	97	90
read-4	gene-3	100	100
read-5	gene-1	95	80
read-5	gene-2	100	100

For gene-1: one uniquely mapping read (read-1); two reads having only gene-1 as best match (read-1, read-2); three reads having gene-1 as best match (read-1, read-2, read-3, the latter having also gene-2 as best match); five reads mapping on gene-1, even if not as best match (from read-1 to read-5)

In our approach, we start from the assumption that the higher is the accuracy of the mapping, the higher is the possibility that read actually originates from such gene. Therefore, if a read maps to two genes with the same accuracy, we consider – without any statistical assumption – that it is equally possible for the read to originate from any of such genes. On the other hand, if a read is more similar to a gene than to another, then it is more possible that the read originates from the former than from the latter, yet without excluding this eventuality (because a low similarity can be due to errors in sequencing or variations in the genome of the sample).

We easily introduce these concepts in our method by exploiting the possibility distribution emerging from the representation of read count through fuzzy sets. If we can define a numerical score to evaluate the accuracy of the mapping results and if we can scale it between 0 and 1, we can exploit it as description of the possibility of reconstructing the right mapping. For example, in NCBI Blast results [20], the numerical score could be defined by the identity of the mapping or by the product of identity and coverage (bit score); in a SAM file the 5th column, which represents the mapping quality between 0 and 255, can be scaled and used as a possibility value. After this computation of possibilities, we obtain a table like Table 2, in which a possibility value is associated to each mapping.

**Table 2:** Possibility values of mappings

Read ID	Gene ID	Mapping possibility
read-1	gene-1	1
read-2	gene-1	1
read-2	gene-2	0.792
read-2	gene-3	0.76
read-3	gene-1	1
read-3	gene-2	1
read-4	gene-1	0.864
read-4	gene-2	0.873
read-4	gene-3	1
read-5	gene-1	0.76
read-5	gene-2	1

In this example the possibility values are computed by scaling the product of Identity and Coverage obtained in Table 1 in a value from 0 to 1

**Fig. 1:**
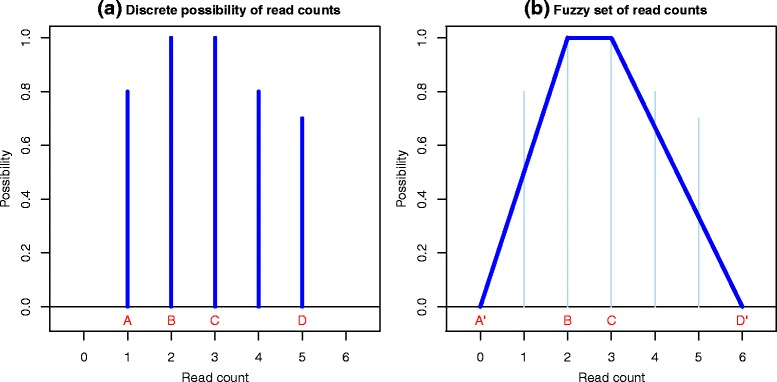
Discrete possibility distribution of read counts. For each gene (**a**) can be represented with a trapezoidal fuzzy set (**b**)

For each gene, we can now compute the possibility of having a given read count. For example, the possibility of having a read count = 1 for *gene-1* is the best possibility of having one read as true match and all the others as false match. In this way, we compute for *gene-1* the possibility distribution shown in Fig. 1. This figure is a fuzzy set, and its interpretation is that the real read count for the gene can possibly be 2 or 3, but it has also an intermediate possibility of being 1, 4 or 5. This is just a representation of what we observe from mapping results, without assumptions on the shape of the distribution and without probability evaluation. The computation of such a fuzzy set is cumbersome; however some general considerations can be drawn to obtain an effective approximation.

In fact, we can observe that the lowest possible value – denoted with an A in Fig. 1(a) – is the number of reads uniquely mapping to *gene-1* and the highest value (D) is the total number of reads that map to that gene (counts outside this interval have null possibility). The plateau with highest possibility is delimited by two other values: the first (B) is the number of reads having the gene as unique best match, the second (C) is the number of reads having that gene as non unique best match (for example read-3 in Table 1 maps both to gene-1 and gene-2 with highest similarity).

We also observe that the possibility value always increases between A and B and decreases between C and D. We can use these four parameters to obtain a simplified representation of the granular read count through a trapezoidal fuzzy set, exemplified in Fig. 1(b) and formally defined as:

1 $$ \mathrm{T}\mathrm{r}\left[\mathrm{A}\hbox{'},\mathrm{B},\mathrm{C},\mathrm{D}\hbox{'}\right](x)=\left\{\begin{array}{c}\hfill \begin{array}{cc}\hfill 0,\ \hfill & \hfill \mathrm{x}\le \mathrm{A}\hbox{'}\vee \mathrm{x}\ge \mathrm{D}\hbox{'}\hfill \\ {}\hfill \frac{\mathrm{x}\hbox{-} \mathrm{A}\hbox{'}}{\mathrm{B}\hbox{-} \mathrm{A}\hbox{'}},\hfill & \hfill \kern1.9em \mathrm{A}\hbox{'}<\mathrm{x}\le \mathrm{B}\hfill \end{array}\hfill \\ {}\hfill \begin{array}{cc}\hfill 1,\hfill & \hfill \kern1.9em \mathrm{B}<\mathrm{x}<\mathrm{C}\hfill \\ {}\hfill \frac{\mathrm{x}\hbox{-} \mathrm{D}\hbox{'}}{\mathrm{C}\hbox{-} \mathrm{D}\hbox{'}},\hfill & \hfill \kern1.9em \mathrm{C}\le \mathrm{x}<\mathrm{D}\hbox{'}\hfill \end{array}\hfill \end{array}\right. $$

where A′ = A-1 and D′ = D + 1 to give non-null possibility to counts A and D respectively. The support of the fuzzy set (defined as D’-A’) quantifies the uncertainty in the evaluation of the expression value, which in turn generates uncertainty in differential expression evaluation. The core of the fuzzy set (defined as C-B) represents the interval with the maximum possibility of containing the real read count.

This kind of representation summarizes the computation of the read count possibility distribution, and it allows to define the fuzzy read counts for each gene with the only computation of parameters A, B, C and D.

### Comparing two conditions

The aim of DE analysis is comparing two or more experimental conditions in order to highlight events in which gene expression presents significant variations, correlated to the experiment. Here we will consider the most common DE analysis, the case–control study, in which the experiment is built assuming that the different conditions between case and control influences only a minority of genes.

**Fig. 2:**
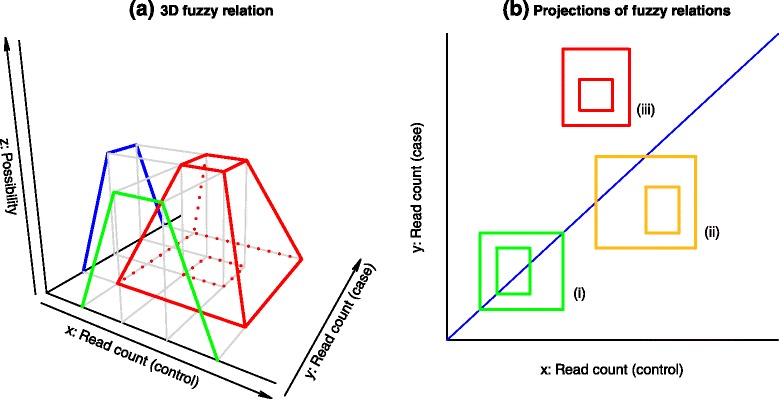
Graphical interpretation of the fuzzy sets and their comparison for differential expression evaluation. The joint possibility distribution of the same gene in two samples is represented by a Cartesian product (**a**). The projections on a 2-dimensional plots can be exploited to evaluate the results (**b**). The green gene (i) has same-expression possibility = 1; the yellow gene (ii) has under-expression possibility = 1, same-expression possibility < 1; the red gene (iii) has over-expression possibility = 1, same-expression possibility = 0

Figure 2 shows a graphical interpretation of comparing fuzzy read counts for the same gene in a case versus control study. Two trapezoids representing the expression of the same gene in different samples can be plotted on a 3-dimensional graph, which is useful to fully understand the use of fuzzy sets and the related possibility distributions. The count values for the two experimental samples are drawn on the *x-*axis and *y-*axis respectively, while the possibility degrees are represented on the *z-*axis. As shown in Fig. 2(a), the Cartesian product of two trapezoidal fuzzy sets, representing the expression of the same gene in different samples, yields a 3D fuzzy relation with the shape of a truncated pyramid. The *z*-value of the pyramid is the possibility degree that the first sample has *x* reads and the second sample has *y* reads for the gene under consideration. We are assuming that the two read counts are non-interacting, i.e. the actual count in the case does not modify the possibility distribution of counts in the control. This enables the representation of the joint possibility distribution by a Cartesian product.

Figure 2(b) shows that the tridimensional figure can be summarized by projecting the two rectangles that contain the support and the core of the fuzzy relation. Larger rectangles represent wider uncertainty, small rectangles (possibly degenerating to a single point) represent more definite results. The position of the rectangle with respect to the bisector line is indicative of the differential expression result in the case–control comparison, but a computational method is required for the analysis of a whole dataset.

First of all, we include in our method the fold change (FC), a widely used metric for DE analysis. The FC is computed as the logarithmic ratio between case and control expression, and it gives an intuitive value of how much the expression has changed between the conditions, independently of the type of normalization applied to the counts.

Through the application of the extension principle, we can extend the definition of FC to granular read counts. For ease of computation we approximate the fuzzy FC by a trapezoidal fuzzy set defined as:

2 $$ \mathrm{T}\mathrm{r}\left[{ \log}_2\frac{A{{\textstyle \hbox{'}}}_1}{D{{\textstyle \hbox{'}}}_2},{ \log}_2\frac{B_1}{C_2},{ \log}_2\frac{C_1}{B_2},{ \log}_2\frac{D{{\textstyle \hbox{'}}}_1}{A{{\textstyle \hbox{'}}}_2}\right] $$

The shape of this fuzzy set gives information about DE of the gene: the wider the range of the trapezoid, the more uncertain is the result; the farther the fuzzy set from FC = 0, the more significant is the DE event.

However, the FC alone is not adequate for the study of DE because it is a ratio, and it is influenced by the magnitude of its terms. The variability observed on gene expression values is amplified by the FC measure for the smaller counts.

**Fig. 3:**
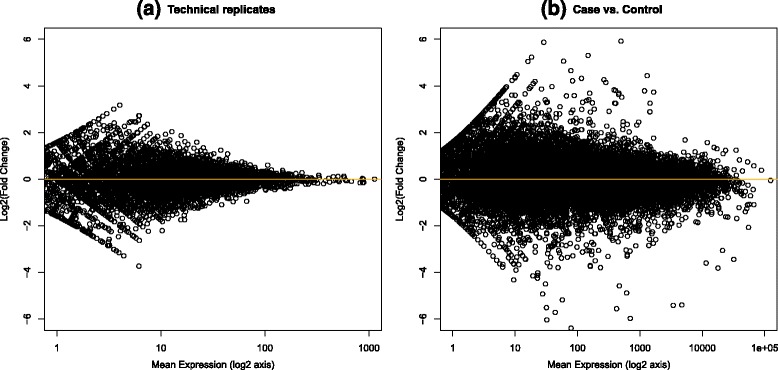
Variation of fold change results correlated to the expression value. The scatter plots show the variability of log2 fold change results for low expression values, in the comparison between two technical replicates of the same sample (**a**) and between two samples in a case-control study

This trend can be studied by analyzing Fig. 3. If we compare two sequencing runs performed on the same sample, namely two technical replicates, we obtain the plot in Fig. 3(a). The scatter plot has on the *x-*axis the logarithmic mean expression value of each gene in the two samples and on the *y-*axis its logarithmic FC value. Logarithm is used on the *x*-axis to draw a more compact image. For ease of representation, only a centroid is represented for uncertain read counts, while the real figure for them would be a kind of tridimensional truncated pyramid. The resulting distribution of data has a roughly rhomboidal aspect: the left part of the rhomboid is shaped by counts near 0, while the right part encloses an area in which the FC reveals a high variability that decreases, as expected, with the increase of mean gene expression. Figure 3(b) shows the same scatter plot for a true case-comparison study: here the DE events are visibly outside the rhomboidal figure.

### Extraction of DE events

**Fig. 4:**
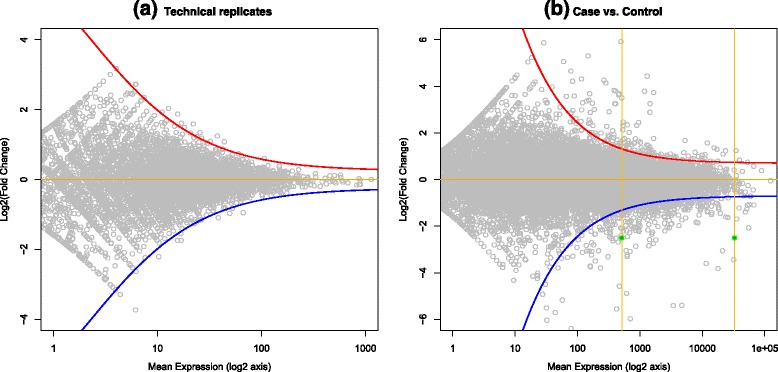
The delimiters for fold change variability. We estimate the borders of the left part of the rhomboid by fitting on the data two hyperbolas. In the comparison between two technical replicates, the curves enclose almost all the points (**a**), while between two samples in a case-control study, the differentially expressed genes fall outside the boundaries. These curves act as a fuzzy threshold for the significance DE events

**Fig. 5:**
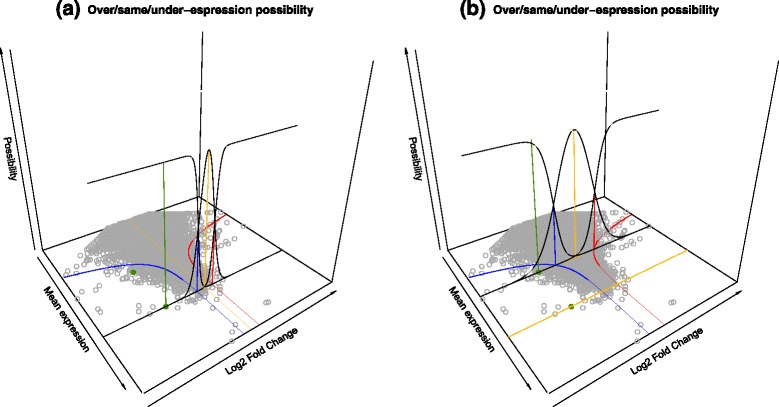
Fuzzy sets for over/same/under-expression possibility. In these examples, we compute the three values of expression possibilities for two points with the same fold change and a different mean expression value. The intersections of the fuzzy sets are defined by the projection of the points on the curves defined in the previous figure, and a lower expression value corresponds to a wider same-expression fuzzy set (the Gaussian membership function)

The DE analysis method proposed for the fuzzy read counts takes in account all the aspects described so far. We estimate the borders of the left part of the rhomboid by fitting on the data two hyperbolas (the curves in Fig. 4, see details in Supplementary information (Additional file 1). They act as a fuzzy threshold for the significance of the fold change, which is as smaller as the mean expression value increases. Each point in the plot corresponds to a gene with a certain mean expression between case and control and the computed FC value. By projecting the point on the *x*-axis, we intersect the two hyperbolas on two symmetric FC values. We use this two values as thresholds points for the definition of three fuzzy concepts for DE: over-expression, under-expression and same-expression. For this purpose, given a mean expression value, we define two sigmoidal and one Gaussian membership functions on the *z*-axis, covering the FC values in order to intersect the two threshold points with membership degree 0.5 (see Fig. 5, for two examples of the definition of the three fuzzy concepts for DE). The Gaussian fuzzy set defines the possibility distribution that a gene is not differentially expressed between case and control (same-expression). The sigmoidal functions describe the possibility of a gene to be over-expressed or under-expressed. The Gaussian and the sigmoidal functions were chosen because they have an infinite support and they are able to describe a gradual increase/decrease of possibility for the FC. In fact, the only aim of these functions is to convert the boundaries defined by the hyperbolas into fuzzy boundaries for the evaluation of the FC values.

**Fig. 6:**
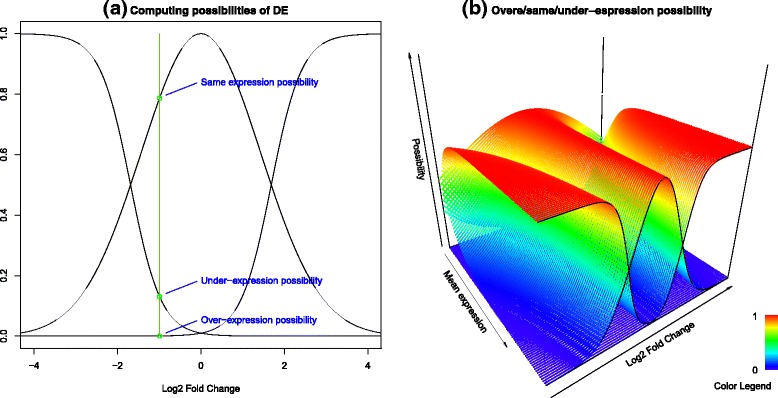
The sigmoidals and Gaussian fuzzy sets and their surfaces. Given a mean expression value, two sigmoidal and one Gaussian fuzzy sets are defined as in figure (**a**). On varying the mean expression value, the membership functions draw three surfaces in the 3-dimensional space (**b**)

When the expression of a gene does not involve multireads, it coincides to a point in the scatter plot of FCs, and its punctual FC value can be directly matched with possibility values from the three fuzzy DE sets corresponding to its mean expression value (Fig. 6(a)). On varying the mean expression value, the 0.5 thresholds are defined on different FC values, following the hyperbolas and the three fuzzy DE sets draw three surfaces in the tridimensional space that are visible in Fig. 6(b).

When the expression of a gene is defined through the fuzzy read count representation, it defines a more complex fuzzy relation on the scatter plot, with a shape similar to a truncated pyramid. In this case the three possibility values of the gene – of under/same/over expression – are obtained by intersecting the tridimensional fuzzy relation of an uncertain gene expression with each of the three surfaces representing the DE possibilities for that gene. The intersection is implemented by the *min* operator and the resulting DE possibility value is the *max* value obtained in the intersection.

Once computed the three DE possibility values, we can evaluate the variation of expression of a gene. A gene with a relevant change in expression will show a high possibility of over or under-expression, but when this result is accompanied by a high possibility of same-expression, we are facing with the eventuality of a false positive result. This DE analysis method is proposed only for case-comparison studies, but it can be applied both in absence and in presence of biological replicates. In the latter case, the set of Gaussian and sigmoidal functions still holds, while the introduction of replicates is included in the fuzzy read count model (described below).

### Result table

The three DE possibility values described are the main output of the proposed method. As a result of the computation we obtain the following values for each gene:

- fuzzy read counts for case (normalized), 4 values defining A,B,C and D points of the trapezoid;
- fuzzy read counts for control (normalized), 4 values;
- fuzzy fold change, 4 values;
- DE possibilities of under/same/over expression, 3 values;
- centroids of the fuzzy sets (for counts and fold change).

In order to provide the user also with a punctual estimation of gene expression in presence of multireads (useful for the plots, for example), we also provide our computation of the centroid of fuzzy sets. For the fuzzy read counts, such values are computed with our “rescue-like” method, a technique based on a modified version of the Rescue Method [9]: while the original model assigns multireads to genes proportionally to the expression of uniquely mapping reads, we assign them proportionally to the score obtained in the mapping. This ensures us to obtain an estimation that is properly centered in our trapezoid, and that respects the proportions of reads of the sample (if we sum the centroid values we obtain the total number of mapping reads). Finally, we provide a punctual fold change value computed on the centroids.

### Normalization

In order to compare two or more samples, we need to normalize the expression values. Many type of normalization have been proposed for RNA-Seq data, and here we adopt the normalization proposed by DESeq2 [6], in which each sample is scaled according to a model based on the geometric mean of values obtained for each gene across all the samples. Applying this type of normalization requires punctual values, so we can consider the genes with only unique matches if multireads are sporadic. If the presence of multireads is important, we can compute a centroid of fuzzy read counts (for example with the “rescue-like” method previously introduced) and use such values for the normalization. Once the scaling factor has been computed for each sample, we can normalize the fuzzy sets by dividing the four defining points of the trapezoids by the scaling factor.

### Introduction of biological and technical replicates

Technical replicates must be merged and considered as a single experiment. As in classic read counting, the technical replicates can be merged before or after the mapping step, and the counts can be merged by sum. This can be done also with fuzzy gene counts, by simply summing the four values obtained for each gene:

3 $$ \mathrm{T}\mathrm{r}\left[{\mathrm{A}}^{\hbox{'}}+{\mathrm{A}}^{\hbox{'}\hbox{'}},{\mathrm{B}}^{\hbox{'}}+{\mathrm{B}}^{\hbox{'}\hbox{'}},{\mathrm{C}}^{\hbox{'}}+{\mathrm{C}}^{\hbox{'}\hbox{'}},{\mathrm{D}}^{\hbox{'}}+{\mathrm{D}}^{\hbox{'}\hbox{'}}\right] $$

where Tr[A′,B′,C′,D′] and Tr[A″,B″,C″,D″] are fuzzy read count for the same gene in two technical replicates.

Biological replicates are different samples belonging to the same condition. This case requires a more reasoned approach, but, as a preliminary method, we propose to merge the fuzzy sets in order to cover all the possible values:

4 $$ \mathrm{T}\mathrm{r}\left[ \min \left(\mathrm{A}\hbox{'},\mathrm{A}\hbox{'}\hbox{'}\right), \min \left(\mathrm{B}\hbox{'},\mathrm{B}\hbox{'}\hbox{'}\right), \max \left(\mathrm{C}\hbox{'},\mathrm{C}\hbox{'}\hbox{'}\right), \max \left(\mathrm{D}\hbox{'},\mathrm{D}\hbox{'}\hbox{'}\right)\right] $$

where Tr[A′,B′,C′,D′] and Tr[A″,B″,C″,D″] are fuzzy read count for the same gene in two biological replicates. When the two fuzzy read counts overlap, the resulting trapezoid includes all the values, by widening its uncertainty range. When the two fuzzy read counts are disjoint, we propose to consider as totally possible even all the intermediate values lying between the observed values. This proposal is based on the following biological interpretation of the data.

The expression of a gene is something that can vary in time. If in the same cell the expression of a gene varies from *N* to *M* between *t1* and *t2*, its variation is not instantaneous (discontinue), but it changes gradually as the copies of the gene are produced (or degraded). This means that between *t1* and *t2* we have a maximum possibility of observing the intermediate values between *N* and *M*. This is true for the same cell and it can be extended to non-independent cells like the ones representing the same condition, as the biological replicates do.

## Results and discussion

The method described has been tested on public datasets and compared with other existing tools for gene counts estimations and DE analysis. We have used RNA-Seq experiments produced with Illumina and 454 Roche Sequencers and we have mapped the reads against the Ensembl’s Vega database [21], evaluating the gene expression. Two preliminary studies have been performed in order to better understand the nature of multireads: an evaluation of the presence of overlapping portions among the genes and an examination of the variability of fold change results correlated to the magnitude of gene expression. All the parameters used to run the different tools are described in Supplementary information (Additional file 1).

### Gene overlapping

**Fig. 7:**
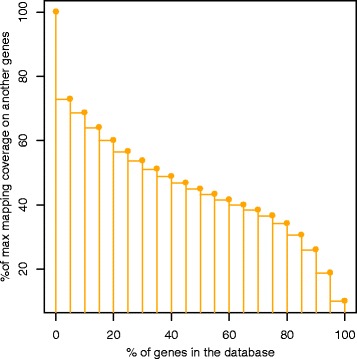
Gene to genes mapping. The result of mapping all the genes against each other reveals that the 50 % of genes of Vega reference database has at least a portion of 45 % of their length in common with at least another gene. The 100 % of genes have a 9 % of their sequence in common with at least another gene

The first preliminary study has been performed on Human reference sequences in order to inspect the presence of overlappings and similarities among genes. Each gene has been mapped against all the other genes present in Vega database. Since a local alignment has been adopted, a 100 % coverage would mean that the queried gene is totally included in another gene. As summarized by Fig. 7, the overlappings are widely present. For example we notice that 50 % of the genes has at least a portion of 45 % of their length in common with at least another gene. In an RNA-Seq experiment, the percentage of multireads is not constant, but it depends on the set of genes involved in the expression profile of the sample and on the accuracy of the sequenced reads.

### Fold change evaluation

The second preliminary study has been performed with the aim of analyzing the range of variability of the fold changes correlated to the mean gene expression value, and the corresponding variation in the number of multireads. This study has been performed through the comparison of two technical replicates obtained by sequencing the same sample two times with the Roche 454 sequencer. We have already shown in Fig. 3(a) the plot of the data for this experiment: the fold change has a wider variation on low expression value that decreases with the increase of expression values. Since this result has been obtained on technical replicates, this means that such variation of the fold change is primarily due to the technical variability of sequencing data.

If we apply our fuzzy method to this data, we can compare the identified trapezoids of fuzzy read counts, with the aim of analyzing if the multiread presence is affected by the technical variability of sequencing data. We notice that 83 % of 19,669 identified genes have a variation in the width of the plateau (the portion between B and C in the trapezoid) of less than 10 reads, while only 8 genes have a variation of more than 20 reads. This means that the multiread presence seems stable across the genes between two technical replicates and then it does not depend on the technical variability, while it is influenced by the experimental conditions and by the set of expressed genes.

### Multiple Sclerosis dataset

The method presented in this work has been tested on public RNA-Seq data and discussed by comparing the obtained results with other bioinformatics tools. The read count estimations have been computed with TopHat (both with uniquely mapping option and with Rescue Method option) and with RSEM (on Bowtie mapping results). We have also considered the centroids of the fuzzy read counts and the number of uniquely mapping reads only, obtained with Bowtie mappings, just to compare RSEM values with other punctual read count estimations.

The DE analyses were performed on the counts with cuffdiff [5], DESeq2 [6], edgeR [7], and with Fisher’s Exact test adjusted by false discovery rate (FDR) [22].

The tests were performed on Illumina HiSeq 2500 data, downloaded from the study SRP055874 [23] of the SRA repository [24], grouped in two different trials. In the first trial we have used only one sample for condition (no replicates), and in the second trial we have selected four biological replicates present in the SRA study, for each condition (8 samples).

#### First trial

The main aim of this test is to analyze the influence of multireads in read count estimations and in DE analysis results. Read counts have been evaluated by using TopHat mappings, the RSEM estimations, the centroids of the fuzzy read counts, and the uniquely mapping reads obtained with Bowtie.

For the DE analysis, cuffdiff was used only with TopHat mappings, because these tools are parts of a unique workflow. TopHat’s read counts are not provided as output, but cuffdiff provides the FPKMs (Fragments Per Kilobase per Million mapped reads), while DESeq2 requires in input the raw read counts. The other estimations of read counts have been analyzed with DESeq2, but the tool does not provide statistically significant differentially expressed genes because of the lack of replicates (a warning message informs the user). We have then applied the Fisher’s Exact test to the counts and we have adjusted the obtained *p*-values for multiple comparisons with FDR.

The mapping identified 25,918 genes, 36 % of reads were multireads and 82 % of gene counts were influenced by multireads. For each DE analysis performed, we have selected the results with *p*-value < 0.05 and abs(log2(FC) > 0.5 and evaluated the uncertainty of the results with same-expression fuzzy set.

**Fig. 8:**
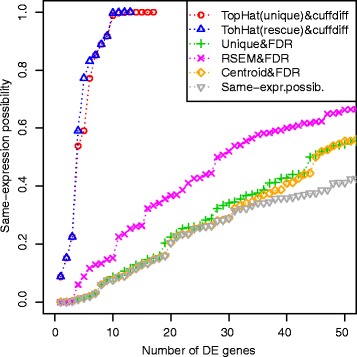
Same-expression possibility in the results of the First Trial. In this trial the results obtained with TopHat show a rapidly increasing same-expression possibility. They have an high possibility of representing false positive results. The plot shows a wide presence of uncertainty in all the results and the grey line of same-expression possibility, by lying below all the other lines, reveals the presence of other differentially expressed genes less influenced by multireads, which have not been discovered by the other tools

The results are summarized by Fig. 8 and Table 3. The figure plots the possibility of same-expression for the genes evaluated as differentially expressed by each analysis. The higher the possibility of same-expression, the more uncertain is the result of the gene, because of the presence of multireads.

**Table 3:** Comparison of the results obtained in First Trial

Results filtered by adjusted *p*-value < 0.05 and abs(log2(FC)) > 0.5	TopHat (unique)& cuffdiff	TohHat (rescue)& cuffdiff	Unique & FDR	RSEM & FDR	Centroid & FDR
TopHat (unique) & cuffdiff	**17**	12	12	13	15
TohHat (rescue) & cuffdiff	12	**13**	11	12	12
Unique & FDR	12	11	**7170**	3224	1244
RSEM & FDR	13	12	3224	**4897**	1381
Centroid & FDR	15	12	1244	1381	**2159**

Each cell represents the number of DE genes in common between two methods specified in the corresponding row and column. Despite the Fisher’s test with FDR extracts a very large list of DE genes, all the results do not agree on at least one gene. The values on the diagonal (in bold) are the results obtained from each method

The uncertainty highlighted in the differentially expressed genes is confirmed by an overall lack of concordance among the DE analyses results, summarized by Table 3. In this table, each cell represents the number of DE genes selected by the two methods specified in the corresponding row and column. Table 4 lists some examples of certain and uncertain results. For each gene in the table we list all the read counts estimations (RPKM for TopHat) and the results of the applied methods.

**Table 4:** Examples of results of the First Trial

GENE: OTTHUMG00000031027|HLA-DRB5					
FUZZY COUNTS	Control	Case	Under-expr.	Same-expr.	Over-expr.
	Tr[18,24,26,428]	Tr[3732,4551,4666,6386]	1	0.003	0
ESTIMATED COUNTS	Control	Case	STATS	*p*-value	Log2(FC)
TopHat-unique (RPKM)	0.2	250.7	cuffdiff	0.9996	10.3
TopHat-rescue (RPKM)	0.2	219.5	cuffdiff	0.9985	10.5
Uniquely mapping	18	3732	FDR	0	7.6
RSEM	23	4665	FDR	0	7.6
Centroid	25	4607	FDR	0	8.1
GENE: OTTHUMG00000036468|TTTY15					
FUZZY COUNTS	Control	Case	Under-expr.	Same-expr.	Over-expr.
	Tr[0,1,1,17]	Tr[43,43,43,52]	0.024	0.986	0.007
ESTIMATED COUNTS	Control	Case	STATS	*p*-value	Log2(FC)
TopHat-unique (RPKM)	0	0.4	cuffdiff	0.0218	Inf
TopHat-rescue (RPKM)	0	0.4	cuffdiff	0.029	Inf
Uniquely mapping	0	43	FDR	1E-14	5.5
RSEM	1	43	FDR	2E-14	4.5
Centroid	1	43	FDR	2E-14	4.9
GENE: OTTHUMG00000129909|IGJ					
FUZZY COUNTS	Control	Case	Under-expr.	Same-expr.	Over-expr.
	Tr[0,0,291,318]	Tr[0,0,1227,1246]	1	1	0.217
ESTIMATED COUNTS	Control	Case	STATS	*p*-value	Log2(FC)
TopHat-unique (RPKM)	16.4	84.9	cuffdiff	0.0218	2.4
TopHat-rescue (RPKM)	17.9	88.4	cuffdiff	0.029	2.3
Uniquely mapping	0	0	FDR	1	0.0
RSEM	250	1165	FDR	2E-208	2.2
Centroid	146	614	FDR	9E-103	2.7

The first gene, HLA-DRB5, despite the abundant presence of multireads in its read counts, shows a certain result, with an maximum under-expression possibility and very low same-expression possibility. This results is not confirmed by cuffdiff, evenif it computes a high FC. The second gene, TTTY15 result is not reliable because it has low read counts, but it is considered as a DE result by all the tools. Our method higlights the result as false positive, with a high same-expression value, because of the low mean expression values obtained. The third example, the IGJ gene, is a possible false positive. Its counts are quite low and its fuzzy read counts are mostly overlapping. The pvalues obtained by the other tools are confirmed by a high under-expression possibility, but the risk of having a false positive is pointed out by a high same-expression possibility

#### Second trial

The same test described for a case-control study with just one sample per condition has been performed introducing biological replicates, downloaded from the same SRA study. We have used four replicates for case and four replicates of healthy controls. In this case the application of DESeq2 to the data gave no warnings and significant DE results, so we have used it for the *p*-value computation of RSEM and rescue-like estimations. Also edgeR has been applied, thanks to the presence of replicates.

**Fig. 9:**
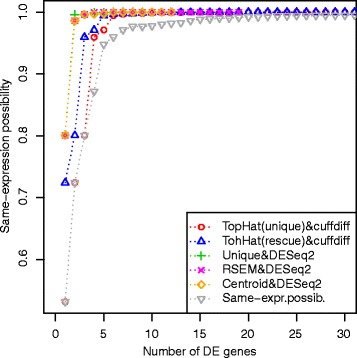
Same-expression possibility in the results of the Second Trial. In this case, there is less difference in the same-expression evaluation of the results obtained with all the tools. The lower value of same-expression obtained on the best result is 0.53

The mapping identified 27,328 genes, 35 % of reads were multireads and 88 % of gene counts were influenced by multireads. The results of the analyses are summarized by Fig. 9 and Table 5. In this case, the presence of biological replicates increases the same-expression possibility of the selected results, and despite the presence of biological replicates, there is still a very low concordance among the various DE analysis results. Multiple Sclerosis is a complex and multi-factorial disease and the introduction of biological variation in this experimentation makes it difficult to extract significant differential expression events. The list of genes selected by at least two workflows, their possibility values and their estimated read counts are showed in Supplementary information (Additional file 1).

**Table 5:** Comparisons of the results obtained in Second Trial

Results filtered by adjusted *p*-value < 0.05 and abs(log2(FC)) > 0.5	TopHat (unique) & cuffdiff	TohHat (rescue) & cuffdiff	Unique & DESeq2	RSEM & DESeq2	Centroid & DESeq2	Unique & edgeR	RSEM & edgeR	Centroid & edgeR
TopHat(unique) & cuffdiff	**56**	36	2	2	2			
TopHat(rescue) & cuffdiff	36	**36**	1	1	1			
Unique & DESeq2	2	1	**9**	7	5			
RSEM & DESeq2	2	1	7	**19**	6		3	
Centroid & DESeq2	2	1	5	6	**12**	1		1
Unique & edgeR					**1**	**1**		1
RSEM & edgeR				3			**4**	
Centroid & edgeR					1	1		**1**

Even in this case, the comparison of DE analyses show a significant disagreement on the selection of DE genes. The values on the diagonal (in bold) are the results obtained from each method

### Simulation study

Since it is impossible to find experiments with information about the real varied and unchanged reads, we have set a simple simulation study to compare the proposed method with other existing tools. We have used *wgsim* by Samtools [25] to simulate 2 experimental conditions with 2 biological replicates, by introducing 10 over-expressed (with log_2_FC = +1) and 10 under-expressed genes (with log_2_FC = −1). The simulated FASTQ files have been processed with TopHat, Cuffdiff, RSEM, DeSEQ2, edgeR and with our fuzzy method. Some examples of the read counts obtained with RSEM and our method are showed in the Supplementary information (Additional file 1).

The results obtained are summarized by Table 6. Cuffdiff with TopHat and DESeq2 applied on the centroids of the fuzzy trapezoids output no significant results (the adjusted *p*-values are > 0.05). DESeq2 applied on RSEM estimation allows to obtain the best sensitivity, while the best recall is obtained by edgeR applied on the centroids. Since our method only ranks the genes, we cannot compare its results in terms of sensitivity and specificity (or precision and recall), and then we will compare the different ranking obtained by all the methods. Anyway, it is interesting to see that, while only 10 genes have a same-expression possibility < 0.5 and all the listed genes have an over- or under-expression possibility very close to 1, the fold change obtained on the centroid values is useful to further evaluate the results. In fact, the expected log_2_ fold changes of about +1 (for over expressed genes) and about −1 (for under-expressed genes) is correctly computed only by our method and by edgeR. DESeq2 always underestimates the fold change. Also Cuffdiff computes a correct fold change for our varied genes, even if it does not consider them significant. The last evaluation is made by comparing the gene ordering given by the same-expression possibility with the sorting of results obtained by the *p*-values of the other workflows. The aim of this evaluation is to see which workflows are able to put the most reliable results in the top of their gene ranking (see Table 7).

**Table 6:** Comparisons of the results obtained on simulated data

Induced variation of expression	Gene	Cuffdiff + TopHat (*p*-value <0.05)	DESeq2 + Centroids (*p*-value <0.05)	DESeq2 + RSEM (*p*-value <0.05)	edgeR + Centroids (*p*-value <0.05)	edgeR + RSEM (*p*-value <0.05)	Same-expression Possibility	Log2 Fold Change on centroids
Under	FADS2			*	**	**	0.000	−1.01
Under	PDE4DIP			*	**	**	0.529	−0.98
Under	U2AF1			*	**	**	0.989	−0.82
Under	WAC			*	**	**	0.000	−1.00
Under	CLCA1			*	**		0.000	−0.97
Under	S100A8				**	**	0.000	−1.04
Under	BPG254F23.5			*			0.889	−0.88
Under	EEF1A1			*			0.889	−0.95
Under	LYZ			*			0.778	−0.91
Under	SCGB1A1						0.000	−0.97
Over	HLA-DQB1			*	**	**	0.778	0.99
Over	IFI6			*	**	**	0.000	0.97
Over	IGF1R			*	**	**	0.000	1.00
Over	MUC5AC			*	**	**	0.000	1.01
Over	POSTN			*	**	**	0.000	1.00
Over	CD177			*	**		0.222	0.99
Over	HLA-DRA			*	**		0.794	0.94
Over	NSUN5P1			*		**	0.889	0.90
Over	UBBP4			*			0.778	0.98
Over	MTRNR2L12						0.778	0.96
None	DUX4L7			*			1	−0.02
None	CH507-152C13.1			*	*		0.995	−0.60
None	AC016698.1			*			1	−0.06
None	MRPL51P2					**	1	−0.05
None	CH507-152C13.4					**	1	0.05
**TP**	**True Positives**	0	0	17	13	11		
**TN**	**True Negatives**	20,000	20,000	19,997	19,999	19,998		
**FP**	**False Positives**	0	0	3	1	2		
**FN**	**False Negatives**	20	20	3	7	9		
**TP/(TP + FN)**	**Sensitivity (recall)**	0 %	0 %	85 %	65 %	55 %		
**TN/(TN + FP)**	**Specificity**	100 %	100 %	99.9 %	100 %	99.9 %		
**TP/(TP + FP)**	**Precision**	0 %	0 %	85 %	93 %	85 %		

This table lists the results obtained on 10 over-expressed and 10 under-expressed genes. Five more genes are involved as false positives. Some workflows select the genes as significant DE events only by the *p*-value (*), while others compute also the correct FC (**). The first two workflows are not able to select significant *p*-values. Ten genes have a low uncertainty (same-expression possibility) and ten genes have a high uncertainty due to their multireads. The five false positive events have a same-expression possibility close to 1 and a non significant FC. Even in this case, there is an overall disagreement in the selection of DE genes. The last 7 rows of the table show some evaluation metrics about the results (Bold)

**Table 7:** Rankings of the genes for the simulated data

Ranking	Same-expression Possibility	Cuffdiff + TopHat (*p*-value)	DESeq2 + Centroids (*p*-value)	DESeq2 + RSEM (*p*-value)	edgeR + Centroids (*p*-value)	edgeR + RSEM (*p*-value)
1	IGF1R	*false positive*	HLA-DRA	PDE4DIP	IGF1R	PDE4DIP
2	POSTN	CD177	MUC5AC	WAC	PDE4DIP	POSTN
3	FADS2	CLCA1	FADS2	IGF1R	WAC	FADS2
4	MUC5AC	EEF1A1	HLA-DQB1	U2AF1	FADS2	IGF1R
5	WAC	*false positive*	WAC	HLA-DQB1	POSTN	IFI6
6	CLCA1	FADS2	IGF1R	FADS2	HLA-DQB1	WAC
7	IFI6	HLA-DQB1	POSTN	MUC5AC	U2AF1	U2AF1
8	S100A8	HLA-DRA	PDE4DIP	POSTN	CLCA1	MUC5AC
9	SCGB1A1	IFI6	S100A8	HLA-DRA	*false positive*	S100A8
10	CD177	IGF1R	CD177	EEF1A1	S100A8	NSUN5P1
11	PDE4DIP	*false positive*	EEF1A1	NSUN5P1	MUC5AC	*false positive*
12	HLA-DQB1	LYZ	CLCA1	*false positive*	HLA-DRA	*false positive*
13	LYZ	MUC5AC	IFI6	CD177	IFI6	HLA-DQB1
14	UBBP4	PDE4DIP	UBBP4	CLCA1	CD177	CD177
15	MTRNR2L12	POSTN	NSUN5P1	LYZ	EEF1A1	HLA-DRA
16	*false positive*	S100A8	LYZ	*false positive*	UBBP4	SCGB1A1
17	*false positive*	U2AF1	MTRNR2L12	*false positive*	SCGB1A1	CLCA1
18	HLA-DRA	WAC	SCGB1A1	IFI6	LYZ	*false positive*
19	*false positive*	*false positive*	U2AF1	BPG254F23.5	NSUN5P1	LYZ
20	*false positive*	*false positive*	*false positive*	UBBP4	MTRNR2L12	EEF1A1

In order to highlight the true positives, the other genes are hidden by a “false positive” label

The worst result is obtained by Cuffdiff on TopHat’s mappings, because it puts a false positive gene at the first position in the ranking. The best result is surprisingly obtained by DESeq2 applied on the centroids of the fuzzy read counts. This workflow did not output significant results but it properly orders the genes, and it puts the first false positive in the 20th position. The second best result is obtained while ranking by same-expression possibility. Here the first false positive result is in the 16th position. Anyway, the good performance obtained by the centroids with DESeq2 can be exploited also to enhance the ranking of the method proposed in this paper. In fact, if we filter the results by fold changes (of centroids) > 0.8, we cleanse the list and we obtain the first false positive in the 20th position.

### Discussion

The tests performed on the selected NGS experiments revealed a high percentage of multireads – 36 % of reads were multireads – that influenced over 83 % of expressed genes. The different approaches used to estimate the read counts have influenced the DE analysis, and all the obtained lists of differentially expressed genes showed some significant differences in the results. In this situation, it is very difficult to perform the choice of a subset of DE events to be confirmed with laboratory assays, such as qPCR. Moreover, since it is impossible to establish which is the right mapping for a multiread, it is also difficult to evaluate the better choice for the estimation of read counts.

Our method is not designed to propose a further estimation of read counts, but it provides a solution for managing the uncertainty in read counts and in DE analysis results due to the presence of multireads. Furthermore, the quantification of under/same/over-expression possibility can be useful in the selection of the more reliable DE events, in order to improve the following laboratory work. The three possibility values can be used just to evaluate the uncertainty of the results of a DE analysis tool, or they can be used in place of it. In fact, a gene with a relevant change in expression will show a high possibility of over or under-expression, and when this result is accompanied by a null possibility of same-expression, we are sure that the multireads are not influencing the result.

The possibility values can be used to properly sort the genes, and we do not propose to apply some thresholds on them, but for an easier interpretation of the results, one could consider an appropriate cut for the discretization of the possibility values and the selection of significant DE events. For instance, by applying a cut of possibility = 0.75 in the results of the first trial of Multiple Sclerosis dataset, we could select 37 DE genes, with the worst same-expression possibility < 0.34. In the analysis of biological replicates of the same datasest, the same-expression possibility is quite high for all the genes. As highlighted by Fig. 9, the lowest same-expression possibility is 0.53. This result can be due to the large amount of multireads found in the experiment, but it might be also caused by the rule that we have chosen to merge the fuzzy read counts of the same gene coming from biological replicates – described in the Methods section – that will be matter of subsequent studies.

## Conclusions

In this paper we have presented a method for dealing with the problem of multireads without statistical assumptions and probability estimations. The method exploits fuzzy sets to describe the presence of uncertainty in read counts estimation and in DE analysis results, whenever the evaluation of the expression of a gene is influenced by multireads.

We have described the trapezoidal fuzzy read counts and fuzzy fold change, and we have modeled the DE events with three fuzzy concepts: over-expression, same-expression and under-expression. The result of the computation is a list of DE events that can be sorted by the possibility of having a change in expression. The DE events are enriched with an evaluation of the possibility of same-expression, that describes the uncertainty of the result due to the presence of multireads mapping to each gene.

In fact, the main aim of this work was to highlight the most reliable DE events and to warn about the more uncertain others, in order to exclude possible false positives from the laboratory assays that follow the bioinformatics analyses.

We have tested the method on some public datasets, and the results revealed that 36 % of the mapped reads were multireads and over 83 % of expressed genes were influenced in their read count by the mapping uncertainty. We have not proved how many DE events that showed high uncertainty are really false positives, because it is very difficult to obtain public dataset in which all DE events have been validated via qPCR, especially because false positive results are not published, usually. Some studies have compared RNA-Seq data with microarray data, but none of the two technologies have been showed to be more reliable than the other on all the genes [26, 27].

In order to give an example of how the multireads influence the discovery of true positive DE events, we have tested the method on a simulated dataset with 10 over-expressed and 10 under-expressed genes. The results showed an overall disagreement in the selection of DE genes, but the proposed method is able to properly rank the most reliable results.

The problem of multireads is frequent in high throughput data analysis and even with longer reads it does not decrease as expected, because there is a high overlapping across genetic portions. We have noticed that in a gene reference database, 50 % of the genes have at most one read that covers a portion of 45 % of their length. The same problem is present in other studies involving read mapping against a reference database. In this paper we have applied our method to gene expression evaluation, but the same concepts can be applied to other studies, such as isoform expression evaluation, ncRNA identification and genomic or metagenomic classification of DNA-Seq reads.

As a future development, we plan to improve the merging of biological replicates step and we will compare our method to other emerging tools. For example, several alignment-free transcript quantification pipelines (e.g., kallisto [28], Salmon [29]) have recently been proposed. These incorporate multi-mapping reads and provide uncertainty estimates for gene counts using bootstrap sampling. Moreover, the same-expression possibility could be directly compared to the output of the DE analysis tool sleuth [30], which evaluates the DE events by considering also the confidence values computed by kallisto.

## Additional file

Additional file 1: Supplementary information about the proposed method and the experimentations performed are provided in “Additional file 1.pdf”. The file is a PDF document and it contains the parameters used for the analyses, the description of the hyperbolic fuzzy boundaries, some additional results on Multiple Sclerosis dataset and simulated dataset. (PDF 642 kb)

## Abbreviations

DE: Differential expression
FC: Fold change
FPKM: Fragments per Kilobase per Million mapped read
NGS: Next-Generation Sequencing
