# Supplementary material for: A fuzzy method for RNA-Seq differential expression analysis in presence of multireads
Source: BMC Bioinformatics. 2016 Nov 8;17(Suppl 12):95–110. doi: 10.1186/s12859-016-1195-2 (PMC5123383; doi:10.1186/s12859-016-1195-2)
Supplement: Additional file 1: — Supplementary information about the proposed method and the experimentations performed are provided in “Additional file 1.pdf”. The file is a PDF document and it contains the parameters used for the analyses, the description of the hyperbolic fuzzy boundaries, some additional results on Multiple Sclerosis dataset and simulated dataset. (PDF 642 kb) [file 12859_2016_1195_MOESM1_ESM.pdf]

A fuzzy method for RNA-Seq differential expression analysis in presence of multireads

Arianna Consiglio[1§], Corrado Mencar[2], Giorgio Grillo[1], Flaviana Marzano[1], Mariano Francesco Caratozzolo[1], Sabino Liuni[1]

[1] Institute for Biomedical Technologies of Bari - ITB, National Research Council, Bari, 70126, IT
[2] Department of Informatics, University of Bari Aldo Moro, Bari, 70121, IT.
[§] Corresponding author

Supplementary information

Parameters used for the analyses

All the dataset used have passed a Quality check performed with the FastQC tool [http://www.bioinformatics.babraham.ac.uk/projects/fastqc/]. All the reads have a good quality and any of them was removed or trimmed.
For the Multiple Sclerosis dataset only the best (in terms of mean read quality) four datasets from case and control were selected for the second trial, in order to limit the complexity of the study and to keep the results as easily assessable by eye. It is known that Multiple Sclerosis is a complex and multi-factorial disease and the biological variability highly influences the uncertainty in the results.
This is the list of the tools used, with their version:

- TopHat v2.0.11
- Cuffdiff v2.2.1
- DESeq2 and edgeR (Bioconductor v.3.2)
- BLAST+ v2.2.28+
- Bowtie2 v2.2.1
- Samtools v0.1.19

All the tools were used with the default parameters, except for the following values: TopHat was used with the option --report-secondary-alignments and Bowtie2 with the -k parameter in order to obtain multireads. Reads mapping to more than 100 different reference sequences were discarded.
Cuffdiff was used with --multi-read-correct option, in order to activate the 'rescue method' for multireads.

Fuzzy boundaries: fitting the hyperbolas

With the aim of defining which areas of the plot include significant DE events and which includes less important variations in expression, two boundaries are defined in the MA-plot. In particular, the borders of the right part of the rhomboid obtained when plotting mean expression versus logarithmic fold change are fitted on the data by using two curves. They provide information to determine the significance of the fold change, following its dependency from the value of the mean expression.
The functions implemented for the curves combine logarithmic and hyperbolic characteristics. It computes the logarithm of a ratio, as in the computation of fold change, and the chosen ratio allows to have two asymptotes: a vertical asymptote on $x = 0$ and a horizontal asymptote on $y = 0$. If we want to exploit the horizontal asymptote as a threshold for the fold change, the constant $c \geq 0$ can be used, otherwise it can be

set to 0. For example, many biologists use 0.6 or 1 as thresholds for interesting $\log_2$ fold change values ($\log_2$ fold change values of 0.6 and 1 correspond to a change in expression of about 1.5 times or of 2 times the expected value). The resulting function is:

$$y = \pm\left(q \log_2\left(x + \frac{1}{x}\right) + c\right)$$

The function is defined for $x > 0$; x represents the mean expression of genes detected in case and/or control (and, therefore, it is strictly positive) and the y represents the logarithmic fold change. The '$\pm$' indicates the sign of the function. For over-expressed data in the upper part of the plot, the relative curve is the one obtained using the *plus*, for under-expressed data in the lower part of the plot, the curve is computed using the *minus*. These curves serve as fuzzy boundaries for DE events, and we adapt them through the parameter q, by using least squares fitting on the most extreme values of the right part of the rhomboid (we are not interested in the left part of the rhomboidal distribution, because it represents genes with too small expressions, anyway those points do not influence the fit because they represent a small percentage of the data). Since the aim of the method is to provide a ranking of the most reliable results in term of increasing same-expression possibility, and in this method the hyperbolas act as a fuzzy threshold for the possibilities, the accuracy of the fit for the hyperbolas is not of primary importance.

Multiple Sclerosis dataset: additional data

The following table shows the genes selected by at least two workflows in the analysis of Multiple Sclerosis dataset.

**Gene**	**TopHat(unique) & cuffdiff**	**TohHat(rescue) & cuffdiff**	**Unique & DESeq2**	**RSEM & DESeq2**	**Centroid &**	**Unique & edgeR**	**RSEM & edgeR**	**Centroid & edgeR**	**Under-expr. Poss.**	**Same-expr. Poss.**	**Over-expr. Poss.**
S100A8	X	X	X	X	X				1.00	0.80	0.00
FADS2	X		X	X	X				0.19	1.00	0.99
HLA-DRA					X	X		X	1.00	1.00	1.00
NCRNA00176			X	X	X				0.01	1.00	0.64
TSHZ2			X	X	X				0.09	1.00	0.98
SMIM1	X	X							0.12	1.00	0.02
IFI6	X	X							1.00	0.97	0.94
DAB1			X	X					1.00	1.00	1.00
S100A12	X	X							0.98	0.96	0.00
HMGN4	X	X							0.21	1.00	0.05
DDX58	X	X							1.00	1.00	0.09
TOR1B	X	X							0.99	1.00	0.07
RNASE6	X	X							0.89	1.00	0.05
HLA-DRB5	X	X							1.00	0.72	0.01
DDAH2				X			X		1.00	1.00	0.99
MDC1				X			X		1.00	1.00	1.00
SIGLEC1	X	X							1.00	1.00	0.45
MPZL1	X	X							1.00	1.00	0.22

ISG15	X	X							1.00	1.00	0.66
AGRN	X	X							1.00	1.00	0.13
MX1	X	X							1.00	1.00	1.00
C21orf33				X			X		1.00	1.00	1.00
RSAD2	X	X							1.00	1.00	0.89
COL18A1			X	X					1.00	1.00	1.00
EIF2AK2	X	X							1.00	1.00	0.09
NFXL1	X	X							0.24	1.00	0.02
MYOM2	X	X							1.00	1.00	0.10
IGJ	X	X							1.00	1.00	1.00
PPBP	X	X							0.97	1.00	0.02
HERC5	X	X							1.00	1.00	0.06
IL1RN	X	X							1.00	1.00	0.04
MYL12B	X	X							0.93	1.00	0.13
TNFAIP6	X	X							0.55	1.00	0.03
CIT	X	X							0.05	1.00	0.10
ARRDC4	X	X							0.97	1.00	0.03
IGHG2	X	X							1.00	1.00	1.00
OASL	X	X							1.00	1.00	0.09
NSUN5P1				X	X				0.88	1.00	1.00
CD86	X	X							0.41	0.99	0.01
CSTA	X	X							0.09	1.00	0.01
BPG246D15.9			X	X					1.00	1.00	1.00
IMMP1L	X	X							0.02	1.00	0.05
CD248	X	X							0.40	1.00	1.00
CADM1	X	X							0.09	1.00	0.09
KRT1	X	X							0.06	1.00	0.99
C12orf42			X		X				0.01	1.00	0.44
C14orf64				X	X				0.01	0.99	0.67
CTC-490G23.2	X	X							0.91	1.00	0.06
CD177	X	X							1.00	1.00	0.44

The following table shows the estimated read counts and the fuzzy read counts of the genes selected by at least two workflows in the analysis of Multiple Sclerosis dataset.

**Gene**	**Unique CTRL**	**Unique CASE**	**RSEM CTRL**	**RSEM CASE**	**Centroid CTRL**	**Centroid CASE**	**Fuzzy count CTRL**	**Fuzzy count CASE**
S100A8	1421	3290	1756	4097	1967	3756	Tr[1421,1421,2448,2448]	Tr[3290,3290,4439,4439]
FADS2	257	155	774	366	798	342	Tr[257,257,1153,1184]	Tr[155,155,575,605]
HLA-DRA	0	784	37	7935	753	3859	Tr[0,0,6516,10313]	Tr[784,1682,13439,14686]
NCRNA00176	18	6	78	11	93	11	Tr[18,22,208,212]	Tr[6,6,22,22]
TSHZ2	78	34	251	87	252	84	Tr[78,78,583,802]	Tr[34,35,155,263]
SMIM1	17	47	40	104	44	95	Tr[17,17,75,75]	Tr[47,47,149,149]

IFI6	860	699	1291	4732	1489	5178	Tr[860,866,2105,2118]	Tr[699,714,15560,15643]
DAB1	53	20	92	39	282	289	Tr[53,57,1288,4913]	Tr[20,25,1958,8163]
S100A12	223	1022	497	1271	540	1184	Tr[223,230,839,839]	Tr[1022,1023,1250,1250]
HMGN4	482	696	628	856	711	774	Tr[482,482,945,946]	Tr[696,696,928,935]
DDX58	777	909	971	1866	1113	1873	Tr[777,782,1282,1310]	Tr[909,914,3621,3662]
TOR1B	794	669	767	1463	865	1486	Tr[794,796,974,978]	Tr[669,669,2809,2815]
RNASE6	245	613	411	944	462	865	Tr[245,245,837,837]	Tr[613,613,1035,1035]
HLA-DRB5	121	3099	681	4673	702	4282	Tr[121,126,2367,3235]	Tr[3099,3779,4776,5873]
DDAH2	0	0	0	255	50	81	Tr[0,0,385,417]	Tr[0,6,731,777]
MDC1	0	0	52	0	47	41	Tr[0,0,664,676]	Tr[0,0,604,612]
SIGLEC1	158	156	320	1394	370	1555	Tr[158,158,576,580]	Tr[156,156,4976,4979]
MPZL1	1012	1921	1622	3561	1805	3254	Tr[1012,1031,2832,2885]	Tr[1921,1952,4491,4532]
ISG15	498	458	652	3034	768	3349	Tr[498,498,1216,1225]	Tr[458,458,10865,10873]
AGRN	33	26	57	128	83	187	Tr[33,33,174,178]	Tr[26,26,743,748]
MX1	1705	1538	2931	9702	3123	10218	Tr[1705,1709,4998,5098]	Tr[1538,1541,29851,29945]
C21orf33	0	2	132	665	331	441	Tr[0,7,1033,1216]	Tr[2,75,1284,1289]
RSAD2	390	301	571	2170	686	2389	Tr[390,390,1150,1153]	Tr[301,301,7656,7658]
COL18A1	196	353	246	498	1145	1605	Tr[196,200,2495,2509]	Tr[353,353,4812,4851]
EIF2AK2	1381	1428	1403	2743	1572	2818	Tr[1381,1385,1906,1937]	Tr[1428,1430,5730,5753]
NFXL1	44	68	60	134	67	130	Tr[44,44,113,115]	Tr[68,68,254,274]
MYOM2	29	29	59	582	66	579	Tr[29,29,148,168]	Tr[29,29,1787,1799]
IGJ	0	0	190	514	113	240	Tr[0,0,404,411]	Tr[0,0,1019,1035]
PPBP	69	144	142	501	155	413	Tr[69,69,202,202]	Tr[144,144,703,703]
HERC5	281	283	334	1179	384	1282	Tr[281,281,475,483]	Tr[283,283,3654,3663]
IL1RN	1748	2128	1912	4169	2142	4270	Tr[1748,1748,2524,2535]	Tr[2128,2133,6348,6352]
MYL12B	599	1455	937	1967	1056	1817	Tr[599,705,1656,2289]	Tr[1455,1714,2055,2673]
TNFAIP6	99	213	175	328	197	320	Tr[99,101,314,330]	Tr[213,214,494,517]
CIT	15	16	17	21	21	20	Tr[15,15,29,147]	Tr[16,16,23,106]
ARRDC4	80	124	144	348	166	351	Tr[80,80,229,229]	Tr[124,124,745,745]
IGHG2	80	108	768	1824	706	1323	Tr[80,159,1717,2063]	Tr[108,262,3774,4330]
OASL	565	539	637	2070	732	2231	Tr[565,568,855,869]	Tr[539,539,5983,5995]
NSUN5P1	65	52	1244	913	1254	793	Tr[65,541,2263,2841]	Tr[52,349,1451,1882]
CD86	289	575	354	754	405	694	Tr[289,291,522,537]	Tr[575,575,782,794]
CSTA	111	166	112	232	129	215	Tr[111,111,146,167]	Tr[166,167,297,305]
BPG246D15.9	24	65	109	380	485	650	Tr[24,24,4599,5366]	Tr[65,65,5571,5765]
IMMP1L	48	29	61	56	71	50	Tr[48,48,110,118]	Tr[29,29,63,75]
CD248	0	0	175	94	108	43	Tr[0,0,534,534]	Tr[0,0,105,105]
CADM1	6	14	13	23	17	22	Tr[6,6,35,136]	Tr[14,14,30,102]
KRT1	134	81	356	205	426	181	Tr[134,134,833,833]	Tr[81,81,297,297]
C12orf42	96	30	125	63	143	54	Tr[96,99,215,353]	Tr[30,31,75,127]
C14orf64	505	358	657	429	756	390	Tr[505,509,1054,1065]	Tr[358,358,431,432]
CTC-490G23.2	2	0	22	60	26	70	Tr[2,2,44,44]	Tr[0,0,242,242]
CD177	6	3	51	177	48	199	Tr[6,6,158,158]	Tr[3,3,1292,1294]

Simulated study: additional data

The following table shows the read counts obtained for the genes already described by **Errore. L'origine riferimento non è stata trovata.** in the main paper. For these genes the table shows that both RSEM and the centroids provide reliable estimations, despite all those genes are influenced by multireads. For the 5 genes that emerged as false positive results when estimated with RSEM, we notice that this tool sometimes is not able to correctly estimate the expression because of the lack of uniquely mapping reads. For simplicity, the mean values obtained for the two replicates are shown for RSEM and Centroids, while the Fuzzy read counts have been merged as described in the Methods.

Induced variation of expression	Gene	TRUE read count for CONTROL	RSEM (CONTROL)	Centroid (CONTROL)	Fuzzy red count (CONTROL)	TRUE read count for CASE	RSEM (CASE)	Centroid (CASE)	Fuzzy red count (CASE)
Under	FADS2	1616	1585.2	1597	Tr[1580,1593,1600,1658]	808	791.8	795	Tr[784,791,798,850]
Under	PDE4DIP	7174	7014.4	6257	Tr[2905,5353,7218,7882]	3587	3525.2	3170	Tr[1482,2688,3681,4366]
Under	U2AF1	1761	1368.3	1123	Tr[508,580,1668,1836]	880.5	557.0	634	Tr[264,289,984,1173]
Under	WAC	2316	2278.3	2283	Tr[2273,2277,2291,2484]	1158	1127.7	1142	Tr[1136,1138,1145,1341]
Under	CLCA1	236	227	232	Tr[230,231,234,235]	118	118	118	Tr[117,118,118,119]
Under	S100A8	124	123.5	124	Tr[122,123,124,125]	62	59.5	60	Tr[58,59,60,61]
Under	BPG254F23.5	280	132.4	40	Tr[0,35,50,297]	140	56.9	21	Tr[0,18,30,204]
Under	EEF1A1	1008	994.8	823	Tr[415,667,1056,1930]	504	483.5	426	Tr[215,340,561,1425]
Under	LYZ	196	197.1	180	Tr[149,156,205,383]	98	95.8	95	Tr[75,79,112,302]
Under	SCGB1A1	66	64.5	64	Tr[62,64,65,66]	33	32.5	32	Tr[31,32,33,34]
Over	HLA-DQB1	2308	981.1	2266	Tr[155,1595,2937,15666]	4616	1918	4506	Tr[280,3147,5865,27821]
Over	IFI6	88	88	88	Tr[87,88,88,89]	176	172	174	Tr[171,172,175,176]
Over	IGF1R	1159	1131.5	1144	Tr[1142,1143,1145,1146]	2318	2262	2284	Tr[2280,2283,2284,2285]
Over	MUC5AC	620	609.5	597	Tr[583,592,602,626]	1240	1213.5	1202	Tr[1176,1197,1207,1231]
Over	POSTN	591	578	586	Tr[583,584,588,589]	1182	1165	1173	Tr[1171,1172,1174,1175]
Over	CD177	152	146.9	147	Tr[96,137,159,184]	304	293.9	292	Tr[183,273,310,335]
Over	HLA-DRA	597	569	587	Tr[0,44,1130,4150]	1194	1153.5	1181	Tr[0,99,2263,8284]
Over	NSUN5P1	446	436.1	448	Tr[55,333,568,1075]	892	889.5	836	Tr[117,682,1009,1540]
Over	UBBP4	76	75.7	76	Tr[16,75,76,316]	152	149.5	150	Tr[18,145,153,417]
Over	MTRNR2L12	37	37.3	37	Tr[1,36,39,121]	74	71.2	74	Tr[0,73,75,148]
None	DUX4L7	45	0	47	Tr[0,0,892,1247]	45	204	46	Tr[0,0,876,1238]
None	CH507-152C13.1	323	287.2	553	Tr[0,10,1099,1185]	323	440.4	364	Tr[0,18,710,758]
None	AC016698.1	32	56.3	32	Tr[0,0,91,150]	32	0	30	Tr[0,0,87,148]
None	MRPL51P2	13	26	14	Tr[0,1,26,34]	13	0	13	Tr[0,0,26,33]
None	CH507-152C13.4	13	0	12	Tr[0,0,25,32]	13	26	13	Tr[0,0,26,33]
